# Jaundice—a Clinical Lecture

**Published:** 1854-03

**Authors:** D. L. M’Gugin

**Affiliations:** Professor of Physiology, Pathology and Clinical Medicine


### *Jaundice—The substance of a Clinical Lecture to the Class Session,* '53–'54.

BY D. L. M'GUGIN, M. D.

Professor of Physiology, Pathology and Clinical Medicine.

The numerous cases which have occurred of this disease, during the past autumn, in the month of October and part of November, in this locality, was the subject of general remark by the profession and demands special attention at this time.

In all the cases the skin was highly colored and after the premonitory symptoms, of which we shall hereafter speak, the icterode appearance would take place very suddenly. The individual would retire for the night without noticing anything peculiar in his appearance, and upon rising in the morning would be surprised, and in some instances, alarmed upon discovering that his face and eyes were stained an intensely yellow color. In a few instances it was confined to the face and neck, in others it would be found upon the entire surface even to the points of the fingers.

It was chiefly confined to adult males, a very few females, and so far as our observation extended, there were no cases in children. It was not preceded or followed by intermittent or any form of fever, nor was it confined to a particular class of persons, for it was observed that those who labored in the outer air were as prone to it as the inactive and sedentary. All temperaments were liable also, but the bilious showed rather more proclivity than the nervous and sanguinous. The greatest distinction was that it was particularly confined to adult males, and the exemption entire on the part of children and an almost entire immunity by the females.

As we have not had reports from other districts, we are not prepared to speak of the extent of its prevalence beyond the limits of this county. Upon enquiry, Dr. Atkinson, of Dover, saw a number, and Dr. Skinner, of Charleston, also saw some cases. Some years since in Autumn, it appeared in parts of Jefferson county, in this State, and from its general prevalence, Dr. Norris, in whose district it prevailed at the time, regarded it as an epidemic. As such it has prevailed in other districts and States of this country.

The great uniformity of the symptoms was a subject of general remark, and the history of the symptoms of one case was but a rehearsal of those of another. For some days preceding the icterode stain there were lassitude, languor, pain in the head, limbs, and back, vertigo, loss of appetite, bitter taste, oppression at the stomach, a dry skin with its temperature diminished, tongue covered with brown fur, bowels constipated, slow breathing, urine frequent, abundant and the color of the infusion of coffee or hops, great depression of spirits, and a cloud dark and gloomy with evil threatnings hanging upon the mind.

To these symptoms in a few days was added the icterode stain, at times light in the shade and anon an intensely dark color and almost black. Generally, in this disease, the natural complexion modifies the shade, but not so in this for the fairer skin became as dark in the shade as in those of a darker complexion, and in these last it was often observed of a bright yellow color.

The general prevalence of the disease, and the great uniformity in the symptoms would point to a generally pervading influence or agent as a cause, and this was doubtless to be referred to a peculiar atmospheric constitution.

A sporadic case of icterus may arise from some hepatic derangement, as congestion, inflammation of the parenchyma of the liver, of the bile ducts, of the duodenum, from the presence of gall stones in the tubuli or the larger ducts which would impair the function of the liver so that the secretion would be arrested, or if secreted, an impediment might arise in its progress through the ducts.

A question of importance in pathology here arises, and which has already given rise to some discussion: and that is, whether bile, once secreted and already in the ducts and cyst and there detained from any cause obstructing its passage, can be again absorbed and returned to the circulation. Some maintain that it cannot, and therefore icterus cannot take place from a re-absorption of that secretion. We cannot fully, or to any extent, subscribe to this doctrine. We maintain that a jaundiced condition may obtain from diseases of the liver and of other structures in close relation, but in such cases it is only symptomatic and wholly dependant upon another lesion of function or structure. In duodenitis and in gastritis it is often an attendant circumstance, and in the former is an almost diagnostic symptom. But it is *only* a symptom, for who would pronounce an inflammation of the duodenum to be icterus.

The disease to which we have called your attention to-day, is idiopathic rather, and there may be an impropriety even in this cognomeral for the primary difficulty may be found in the blood itself. The truth doubtless lies in faulty elimination, but to what are we to attach the blame, or in what does the primary difficulty lie? Is it because the liver refuses any longer to act as the labrator, or is it not rather because of the changed condition of the material from which it withdraws certain products with which to form the bile, and therefore is unable to discharge faithfully its legitimate function? The proximate principles are then retained in the circulation becuse they are not elaborated by the secreting offices of the liver. The position that there is a change in the constituents of the blood is favored by another fact which has been observed, and that is that the morbid anatomy shows the secreting cells to be filled with a black pigment rather than the brown transparency which characterizes this normal condition. This shows an abnormal state of the primary stage of secretion, for no lesion is found to exist in the tubuli or the larger ducts which are without bile.

The coloring matter only is found to pre-exist in the blood, but the other elements only exist as proximate principles, and it is the duty of the liver so to combine them as to form the bile. If these principles do not exist in a normal proportion or a normal condition, healthy bile cannot be formed, or none is formed of any kind.

The coloring matter then is distributed with the other principles, to all parts of the system, the first leaving its traces and marks in the textures, while the latter wreaks its poisonous influences upon the vital organs, and these too in the order of their vital relations. The brain suffers most, and hence the pain in the head attended with vertigo and the great depression of spirits.

Under these circumstances it is necessary that the other depurators should be awakened into activity in aid of the kidneys already spontaneously awakened to increased effort. Fortunately for those afflicted that these important emunctories did take upon themselves an increased duty, in order as far as possible to diminish the morbid accumulations. Acting, however, almost alone, it was not to be expected that they would be competent to the requirements. The loss by the skin, the lungs and the bowels, of this poisonous material rather than being increased was diminished, for their functions were below the healthy standard. It would be difficult to say in these cases how far the kidneys were able to repair the deficiency or make up for the defect of each. There is not a reasonable doubt, however, that their increased activity and efficiency warded off, in a good degree, more serious results.

If this view of its pathology be correct then, the first indication would be to awaken into activity and action the other depurators for the reasons as above stated. Mass hyd in combination with quinia, diaphortics, saline draughts, are those remedies found to be speedily successful. The combination of ox gall inspissated will, with some other aperient, constitute a compound highly valuable in these and all cases of suspended biliary secretions, and when effete matters of the bowels become concrete. The ox bile is not only a substitute for bile, but it possesses the property also of a solvent for alvine concretions.

Let then the skin be awakened to increased action, the bowels to moderate activity, the kidneys encouraged to a perseverance in their good offices and if possible to increase their efforts, induce the liver to arouse to fresh exertion, and soon the lungs will fall in with the general effort to rid the circulation of its deleterious complications and combinations, and in this way an intermittent, a remittent, a continued, or an enteric fever, may be avoided.

In some instances the skin remained stained for a long time after the constitutional symptoms had passed away, but in the sanguine and those of an active capillary circulation, it speedily disappeared.

A popular impression, I am informed by one of my professional brethren, prevails here that there is some relation existing between the Yellow Fever and this epidemic Jaundice. The subject had once been mooted before, and the conclusion was then, that the idea had its origin in the fact of the "yellow stain," and therefore no attention was paid to it. But it is asserted that when the former disease prevailed epidemically and fatally at New Orleans some years since, that Jaundice was extensively prevalent here. If this should prove to be the case it would, to say the least of it, show a singular coincidence. As it is a fact to be established by datta, and as these are now not at hand, we shall institute some further inquiries into the facts, and defer an opinion until then. The single coincident circumstance which occurred this last autumn would not by any means be sufficient to show a relation, or that it was a diffused but modified epidemic influence pervading to this extent.

